# Noninvasive preimplantation genetic testing for aneuploidies (niPGT-A) and the principle of *primum non nocere*

**DOI:** 10.5935/1518-0557.20200075

**Published:** 2020

**Authors:** Jose G. Franco Jr, Felipe Dieamant, Joao Batista A. Oliveira

**Affiliations:** 1 Center for Human Reproduction Prof. Franco Jr., Ribeirão Preto, SP, Brazil

**Keywords:** Noninvasive preimplantation genetic test, niPGT-A, PGT-A

## Abstract

Perhaps with the intention of obtaining larger amounts of free-DNA, some groups are routinely postponing and establishing free-DNA collection in culture medium for Noninvasive preimplantation genetic testing for aneuploidies (niPGT-A) to day 6 for all blastocysts. A meta-analysis served as the basis for such decision, since statistically similar live birth rates were observed when the transfers of euploid blastocysts were performed on day 5 versus day 6 However, the euploidy analysis was conducted in only two studies However, after including the two more studies we performed a new meta-analysis that clearly showed the risks of losing live births with the decision of adopting the 6th day as the endpoint for gathering free-DNA. We would be losing 1.71x more live births.

The development of preimplantation genetic tests for aneuploidies (PGT-A) has a history of over 25 years. Initially, the invasive method labeled ‘preimplantation genetic screening’ (PGS) received its first critiques when Mastenbroek *et al*. (2007) published a randomized controlled study in which PGS was indicated for advanced maternal age. Its use did not increase, but instead, significantly reduced the rates of ongoing pregnancies and live births (PGS=24%; Control=35%). This was the first time in which the use of a PGT (PGS) did not fulfill the principle of primum non nocere. Thus, those who supported PGS started to attribute the burden of negative outcomes to imperfect FISH techniques in chromosome abnormality assessments and the fact that biopsies performed on the third day (embryo cleavage stage) would carry a high frequency of mosaicism. Hence, a new PGS method was developed, with the embryo biopsy on the 5th or 6th day of embryonic development and the use of new chromosome assessment techniques (comparative genomic hybridization-CGH; next-generation sequencing-NGS, etc.) that were safer in establishing chromosomal abnormalities, in addition to enabling the analysis of all 24 chromosomes.

Despite the substantial technological development in this area, doubts arose regarding the invasive approach (known as inPGT-A in recent years) due to the occurrence of a percentage of false-positive diagnoses in the blastocyst biopsy of the trophoblast, especially in situations of embryonic mosaicism. Greco *et al*. (2015) described the first births of healthy children with mosaicism after embryo transfer. This fact alerted experts in the area about the risk of discarding countless embryos with a potential for implantation. It is noteworthy that the percentage of blastocysts with mosaicism does not usually vary with age, although its frequency could reach up to 50% in some laboratories (Popovic *et al*., 2020). This would constitute the second time that a PGT-A (inPGT-A) was disrespecting the principle of primum non nocere.

Xu *et al*. (2016) described the birth of several children using noninvasive PGT-A (niPGT-A) through the collection of free-DNA, which was secreted into the embryonic culture medium during human embryo development from cleavage until the blastocyst stage. By using multiple annealing and looping-based amplification cycles (MALBAC) for whole-genome amplification (WGA), the authors performed NGS on the free-DNA obtained in the spent culture medium of the blastocysts on day 5 (n=42) and were able to analyze all 24 chromosomes. In order to validate their results, they compared the chromosomes in the culture medium with their corresponding whole donated embryos. The authors found a significant correlation in the identification of chromosomal abnormalities (sensitivity: 0.882; specificity: 0.840). With this validated niPGT-A method, they performed chromosome screening on IVF embryos from seven couples with balanced translocation, azoospermia, and recurrent pregnancy loss. As a result, six of them achieved successful clinical pregnancies and healthy live births. This niPGT-A method avoids the need for embryo biopsy and, therefore, substantially increases the safety of its use. The approach has the potential for much wider chromosome screening applicability in clinical IVF on account of its optimal accuracy and noninvasiveness (Fang *et al*., 2019; Jiao *et al*., 2019; Rubio *et al*., 2019; Olcha *et al*., 2020).

Despite this promising start, difficulties in the use of niPGT-A have been reported, which could be solved. In principle, a validation program is mandatory for groups that are interested in conducting niPGT-A before collecting free-DNA in spent culture medium. In such training, the risk of contamination with the patient’s granulosa cells should be discussed with embryologists, and the group should be taught the appropriate measures for denuding embryos to reduce the levels of free-DNA contamination submitted to niPGT-A to below 2%. Currently, some softwares can already use artificial intelligence to detect cases suspected of contamination. In this situation, a new free-DNA collection is required. Also, the technique for collecting free-DNA must be strictly standardized for each laboratory. This includes special pipettes, culture plates suitable for reduced volumes of culture medium, and the determination of use of a sequential or continuous culture system, depending on each laboratory’s routine. All groups submitted to the validation process need to be efficient in free-DNA collection protocols, and the results must be reliable in both fresh embryonic and frozen-thawed cycles.

Regarding chromosomal mosaicism, Vagnini *et al*. (2020) described an incidence rate of approximately 32% in human blastocysts, established by niPGT-A using the NGS platform and the cut-off adopted by specific software. However, it did not vary remarkably with age. Euploidy levels had a negative correlation with increasing age, whereas aneuploidy levels presented a positive correlation with it. Therefore, the careful interpretation of the mosaicism phenomenon, established by niPGT-A, should be a priority to avoid discarding potentially normal embryos. Unfortunately, these guidelines still require clarification. Even when adopting the precautions suggested above, it is important to remember that PGT techniques can select euploid embryos within several embryos with euploidy, aneuploidy, and mosaicism. Nonetheless, euploids fail to implant in 30% to 40% of embryonic transfers. Therefore, the primary goal of laboratories should be to produce more euploid embryos.

On the other hand, perhaps with the intention of obtaining larger amounts of free-DNA, some groups are routinely postponing and establishing free-DNA collection in culture medium to day 6 for all blastocysts (Rubio *et al*., 2020). The meta-analysis published by Bourdon *et al*. (2019) served as the basis for such decision, since statistically similar live birth rates were observed when the transfers of euploid blastocysts were performed on day 5 versus day 6 (analysis subgroup). However, the euploidy analysis was conducted in only two studies (Barash *et al*., 2017; Coates *et al*., 2017). We highlight that the study by Taylor *et al*. (2014) and Irani *et al*. (2018) were not included in this meta-analysis subgroup regarding the transfer of euploid embryos (day 5 versus day 6).

Therefore, after including the data above, we performed a new meta-analysis that clearly showed the risks of losing live births with the decision of adopting the 6th day as the endpoint for gathering free-DNA. We would be losing 1.71x more live births, as shown in Figure 1. Collecting free-DNA on day 5 would be ideal, as long as its development and blastulation are compatible. Collection could also be performed on the 6th day since the embryo reaches blastulation later. However, even if it were euploid, it would produce an important reduction in the percentage of live births. Moreover, when Bourdon *et al*. (2019) evaluated both fresh and frozen-thawed cycles, without genetic analysis, they concluded that ART practitioners should preferably transfer D5 rather than D6 blastocysts.

**Figure 1:**
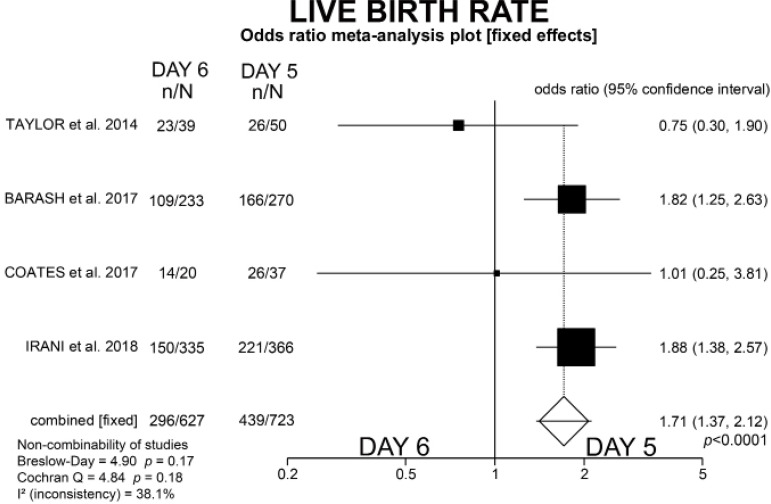
New meta-analysis graphic

In conclusion, establishing the 6th day as a routine for collecting free-DNA for niPGT-A will undoubtedly be the third disobedience of PGT regarding the principle of primum non nocere.
